# Quantifying the Selection of Maxillary Anterior Teeth Using Extraoral Anatomical Landmarks

**DOI:** 10.7759/cureus.27410

**Published:** 2022-07-28

**Authors:** Sweta Pisulkar, Sharayu Nimonkar, Akansha Bansod, Vikram Belkhode, Surekha Godbole

**Affiliations:** 1 Department of Prosthodontics and Crown and Bridge, Sharad Pawar Dental College and Hospital, Datta Meghe Institute of Medical Sciences, Deemed to be University, Wardha, IND; 2 Department of Prosthodontics, Sharad Pawar Dental College and Hospital, Datta Meghe Institute of Medical Sciences, Deemed to be University, Wardha, IND

**Keywords:** esthetics, facial proportion, inter-commissural distance, inter-alar distance, inter-canthal distance

## Abstract

Background: Regardless of skin color, age, sex, or other factors, all beautiful faces follow the divine proportion. Proportions of face components appear to play a role in facial attractiveness.

Aim: The study determines the correlation between the mesiodistal width of anterior maxillary teeth, inter-canthal distance, inter-alar distance, and inter-commissural distance as a criterion for selecting anterior maxillary teeth.

Material and Methods: A cross-sectional study was carried out at the institutional level with a sample size of 200 participants with 100 male and 100 female participants. Inter-canthal distance, inter-alar distance, and inter-commissural distance were measured using the digital caliper and a customized silver scale to measure the mesiodistal width of anterior maxillary teeth. The data obtained were analyzed using Statistical Package for Social Sciences (SPSS) version 24.0 (IBM Corporation, Chicago, USA). Pearson's correlation test calculated the correlation coefficient, and the level of significance was kept at p<0.05.

Result: The width of the maxillary anterior and the inter-canthal gap showed a strong positive correlation, the inter-alar difference and the mesiodistal width of anterior teeth showed a moderately positive correlation, and the estimated inter-commissural width and the mesiodistal width of anterior teeth showed a strong positive correlation (p<0.05).

Conclusion: These parameters are correlated to the dimensions of the tooth size and can act as a guide while selecting the teeth for future rehabilitation treatment when the natural teeth are lost.

## Introduction

Every existence of God in the universe follows the golden proportion of 1:1.618, and the face is no exception. Regardless of color, age, sex, or other factors, all beautiful faces follow the Divine Proportion. Various facial components are in specific proportions, resulting in a stunning look [1]. Aesthetic beauty and facial appearance have a psychological effect on the human personality and human relationships. According to psychologists, more attractive people are more acceptable in society [2].

To achieve an optimal aesthetic result, dental practitioners must determine tooth size and shape when treating patients with missing anterior maxillary teeth [3]. If the size and shape of a replaced tooth are not in harmony with patients' faces and other teeth, psychological and social problems might arise [4].

Dentures should be as natural-looking as natural teeth in completely edentulous patients, so choosing artificial teeth is an essential consideration in complete denture design. The tooth size and form selection are even more complicated for the edentulous patient with no pre-extraction history. A tooth not the same size or shape as the patient's natural tooth will entirely alter their physiognomy.

The anterior maxillary teeth must be proportionate to facial morphology to appear attractive. Several anatomic measurements have been proposed to aid in determining the correct size of the anterior teeth, among them the inter-commissural width, bizygomatic width, inter-alar width, and interpupillary distance [5]. Specific authors have proposed a relationship between the width of the maxillary central incisor and the interpupillary distance [6]. Similarly, a proportional relationship between the widest part of the nose and the anterior dental arch has been reported [7]. These suggestions, therefore, should be validated by additional scientific studies in different populations.

This study will help in the preliminary analysis of the condition by careful teeth selection, thereby preventing distress among the patients and leading to primary care. Hence, the present study aims at determining the correlation between the mesiodistal width of anterior maxillary teeth, inter-canthal distance, inter-alar distance, and inter-commissural distance as a criterion for selecting anterior maxillary teeth.

## Materials and methods

Study design

The study was a cross-sectional study, conducted in the Department of Prosthodontics. The Institutional Ethical Committee obtained ethical clearance to address the proposed study's ethical aspect (IEC No. IEC/DMIMS/2020-21/262). The duration of the study was one year.

Sample size calculation

FORMULA - n = Z21-α/2 σ2/d2, 

where, n = 200 sample size, Z21-α/2 = 80% confidence interval, σ = 12.36 estimated standard deviation, d = 0.5 desired precision.

As per the formula, the sample size was 200, with 100 male and 100 female subjects taken as samples for the study.

Inclusion criteria

Participants in the age group, 18-24 years with all permanent teeth and no dentofacial anomaly, were included in the study. Written informed consent from each participant was obtained.

Exclusion criteria

Participants with artificial crowns on upper teeth. In whom the upper teeth are associated with gingival inflammation, hypertrophy, or orthodontic treatment were excluded from the study.

Calibration of examiners

**Figure 1:**
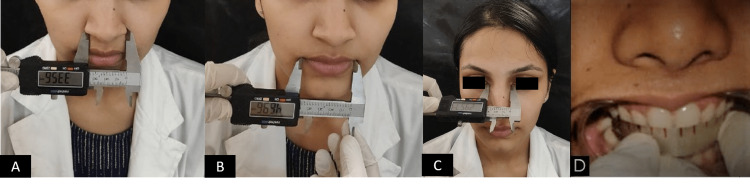
Method of measurement of the following parameters. (A) Inter-alar distance, (B) inter-commissural distance, (C) Inter-canthal distance, (D) mesiodistal width of anterior maxillary teeth.

The subjects in the study were evaluated based on three parameters, inter-canthal distance, inter-alar distance, and inter-commissural length. Intra and inter-examiner reliability (R=0.7) was determined by setting standard landmarks, and repeated measurements and cross-examination were also performed. They were correlated with the width of the maxillary central incisor (Figures 1A-1D).

**Figure 2:**
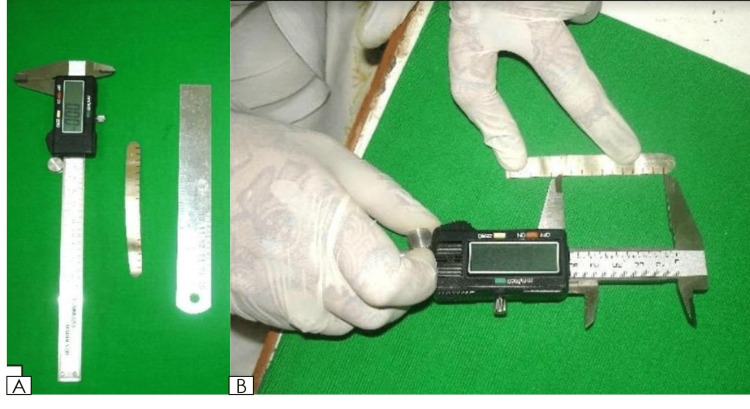
(A) Digital caliper, metallic measuring scales. (B) Measurement transferred to the metallic scale for readings.

The accuracy of the measurements was maintained by cross-examination by other examiners. Each examiner recorded the measurements three times to minimize errors by two examiners. Two examiners followed the same procedure to eliminate the discrepancy. All the examiners were trained in using the digital caliper and the metallic scale (Figures 2A, 2B).

During the measurement, the subjects were seated in the dental chair and asked to respire normally and then hold their breath, as the nares tend to flare while breathing, to ease the anxiety among the participant and reduce labor-intensive errors while measuring the lengths mentioned above.

Intervention

Measurements of the inter-canthal distance, inter-alar distance, and inter-commissural distance were recorded using the digital caliper (0.1 mm precision) (Mitutoyo Digital vernier caliper). A customized silver scale, as this is the most convenient and simple to use the tool, most of the studies have utilized a customized scale, scale recorded the mesiodistal width of anterior maxillary teeth by adapting it to the six anterior teeth starting from the distal contact point of the right maxillary canine to the distal contact point of the left maxillary canine [2]. The widths were measured, and all the information obtained from the evaluation was statistically analyzed. Three readings were recorded for all the parameters, and an average value was considered. The obtained data were compared using statistical analysis.

Descriptive and analytical statistics were done (Statistical Package for Social Sciences [SPSS] Version 24.0 (IBM Corporation, Chicago, USA]). The data were represented in mean and standard deviation. A Pearson's correlation test calculated the correlation coefficient. The level of significance was kept at p<0.05.

## Results

**Figure 3:**
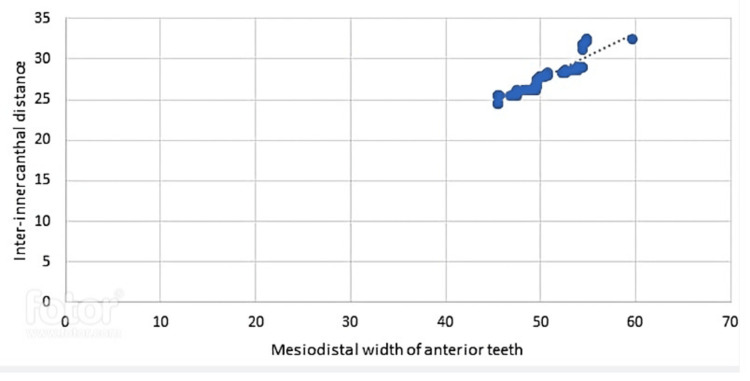
Correlation of inter-canthal distance with mesiodistal width of anterior teeth.

A statistically significant (p>0.001) strong positive association R=0.904 exists between the inter-canthal difference and the mesiodistal width of anterior teeth. This means that the wider the inter-canthal gap, the wider the anterior teeth's mesiodistal width, correlation, and frequency distribution (Figure 3).

**Figure 4:**
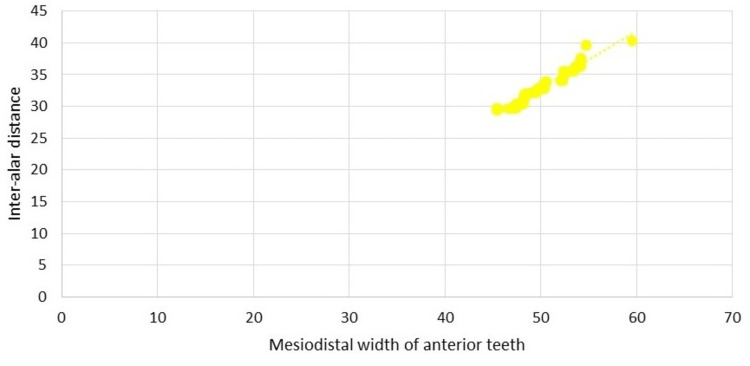
Correlation of inter-alar distance with mesiodistal width of anterior teeth.

The inter-alar difference and the mesiodistal width of anterior teeth had a moderate positive correlation R=0.966 that, was statistically significant (p>0.001), as well as the frequency distribution and correlation. This means that the wider the inter-alar gap, the wider the anterior teeth's mesiodistal width (Figure 4).

**Figure 5:**
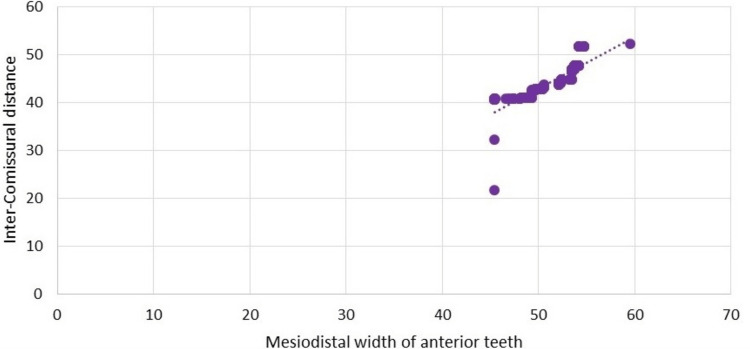
Correlation of and inter-commissural distance with mesiodistal width of anterior teeth.

Inter-commissural distance and mesiodistal width of anterior teeth were found to have a statistically significant (p>0.001) strong positive correlation R=0.848, as well as the frequency distribution and correlation. This means that the wider the inter-commissural gap, the wider the anterior teeth's mesiodistal width (Figure 5).

The inter-canthal gap, inter-alar distance, and, inter-commissural distance variable, differentiated on the basis of gender was insignificant. The study found a strong positive correlation between the inter-canthal gap and the width of the maxillary anterior (p>0.05), a moderately positive correlation with the width of the maxillary anterior, and a strong positive correlation with the estimated inter-commissural width (Table 1).

**Table 1:** Correlation of inter-inner canthal distance, inter-alar distance, and inter-commissural distance with mesiodistal width of anterior teeth #P-value derived from Pearson's correlation test; †significant at p < 0.05

Variables	N	R-value	P-value^#^
Inter-inner canthal distance	200	0.904	<0.001^†^
Inter-alar distance	200	0.966	<0.001^†^
Inter-commissural distance	200	0.848	<0.001^†^

## Discussion

Dentures aid in the restoration of a fully edentulous patient's appearance. The harmonization and correlation of replacing single or multiple teeth with the adjacent anatomical structures are also necessary in cases of partially missing teeth. Since there are no pre-extraction data, choosing the dimension and shape of artificial teeth is difficult. Varied methods for measuring the size of artificial teeth in the dental literature are available. However, no universally accepted protocol exists at this time. Most techniques are based on soft tissue landmarks, and age-related variations in soft tissue measurements have also been identified [8].

Recent scientific literature reviews studies that were carried out using different methodology and sample sizes, different face and natural teeth parameters [9,10] casts, [11,12] or photographs, [13,14] as well as various types of gauges which make the comparison of results very difficult. The instruments varied from traditional Boley's gauge, digital Vernier calipers, Willis Gauge, War wins foldable scale, modified dividers, and flexible rulers to image processing software (HL Image). Varejao et al. used a vernier caliper to measure inter-alar and intercanthal widths [10]. The present study used a digital caliper with a modified metallic scale for all the variables.

All the variables, inter-canthal, inter-alar, and inter-commissural distance, were significantly correlated to the width of maxillary incisors. Various studies are mentioned in the literature with similar correlations used for teeth selection in wholly and partially edentulous patients. These suggest using different anatomical landmarks that act as reference guides for teeth arrangement and artificial teeth replacement, such as the incisive papilla, the contour of the residual alveolar ridges, etc. A study conducted by Patel et al. in 2011 involving 240 participants found that when multiplied by a ratio of 1.61 and 1.45, the Inner-canthal distance or the inter-alar distance could also be used as a preliminary indicator for the estimate of the inter-canine width of the maxillary six anterior teeth [15]. These findings are in collaboration with our study, which suggests that intercanthal distance was correlated to the width of anterior maxillary teeth.

The systematic review conducted by Jain et al. aimed to be the most reliable indicator of the various anthropometric measurements in determining the width of anterior maxillary teeth in different populations [16]. The study revealed a high degree of correlation between inter-alar width, interpupillary distance, bizygomatic width, and width of anterior maxillary teeth in the Indian population. Esan et al. found that in the Saudi people, intercanthal distance showed a high correlation to the width of anterior maxillary teeth. In the Brazilian (mulatto and blacks) population, a high correlation was seen between inter-commissural width and width of anterior maxillary teeth [17].

Therefore, these parameters are correlated to the dimensions of the tooth size and can act as a guide while selecting the teeth for future rehabilitation treatment when the natural teeth are lost. These guides need not be preserved as we preserve the patient's pre-extraction records for future reference. Various studies are mentioned in the literature with similar correlations used for teeth selection in partially and completely edentulous patients [18-20].

While the preserved photographs of the patient can be used as a reference for tooth selection and arrangement calculations, there are many instances where the teeth are not apparent in the snapshot, or the measurements are too small to be calculated for a meaningful result. As a result, specific anatomical landmarks are also used to estimate the size of the teeth that will be chosen. Other experiments that compared variables discovered that the distance between the tips of the upper canines and the widths between the ala of the nose has the most substantial relationship [7].

The maxillary central incisor is a good reference point for artificial tooth placement. According to research, the distance between the tips of the upper left and right canines is the same as the distance between the alae of the nose. In a few studies, nasal width has been suggested as a predictive factor [9]. Smith studied the relationship between inter-canine width and the bony aperture of the nose on face roentgenograms of the head. He discovered that the differences between the two widths varied in a range of 0.2 mm to 5 mm [18].

Apart from previous registration and plaster cast development of a person's teeth, or if we have the extracted teeth of any individual, none of the theories on the choice of the size or shape of artificial teeth still fulfill all requirements; they only approximate the values required [21]. As a result, the anatomical reference that remains with the patient as a reference is a viable choice.

The present study has determined the correlation between the anterior maxillary teeth' mesiodistal width, inter-alar distance, and Inter-commissural distance as a criterion for selecting anterior maxillary teeth. A strong positive correlation was obtained between the inter-canthal distance and the inter-commissural distance and a moderate correlation between inter-alar and the width of anterior maxillary teeth. These findings help quantify the selection of teeth using the extraoral anatomical landmarks. Correlating the landmarks to the width of anterior teeth helps relieve the distress among the patients and is a guide to dental professionals in selecting anterior teeth, which in turn is ultimately helpful in primary care. Small sample size remains the major limitation of the present study; moreover, the subjective bias in determining the inter-canthal distance, inter-alar distance, and inter-commissural distance may also lead to a false interpretation of the values.

## Conclusions

The anterior maxillary teeth must be proportionate to the facial morphology to be attractive. The study found a strong positive correlation between the inter-canthal and inter-commissural distances and a moderate correlation between the inter-alar and maxillary anterior tooth width. Inferences drawn from these landmarks during teeth selection would guide dental professionals in making appropriate choices. Precise teeth selection would aid in achieving pleasing aesthetics, relieving patients' anxiety about their appearance and facial aesthetics. In the present study, measurements were made manually and later the correlation was interpreted in teeth selection.

In older times, gothic arch tracing was used to determine teeth size, but it was subjective guidance recently with the evolution of more stable anatomic landmarks. Through the use of these landmarks, findings could be made more objective. Furthermore, the current digital approach could record the measurements with the help of custom-designed software. This technological advancement could relate to adding up the facial aesthetics and assisting in making the appropriate choice for each individual.
